# Scimitar Syndrome in Adulthood: Challenges in Management and Individualized Approaches

**DOI:** 10.7759/cureus.61857

**Published:** 2024-06-06

**Authors:** Milan Luknár, Peter Hlivák, Peter Lesný, Eva Goncalvesová

**Affiliations:** 1 Department of Cardiology, Faculty of Medicine, Comenius University, Bratislava, SVK; 2 Department of Cardiology, Faculty of Medicine, National Cardiovascular Institute, Bratislava, SVK; 3 Department of Arrhythmia and Pacing, Slovak Medical University, Bratislava, SVK; 4 Department of Arrhythmia and Pacing, National Cardiovascular Institute, Bratislava, SVK; 5 Department of Cardiology, Faculty of Medicine, Comenius University and National Cardiovascular Institute, Bratislava, SVK

**Keywords:** abnormal pulmonary vein, congenital disease, stenosis, intervention, adult, scimitar syndrome

## Abstract

Scimitar syndrome is a congenital disorder characterized by partial anomalous pulmonary venous return to the inferior vena cava (IVC). Clinical manifestation in adulthood is infrequent. The management approach has not been universally accepted and may be challenging. Individually tailored and multidisciplinary team-based decisions are often necessary. We present the case of a symptomatic patient diagnosed with complex congenital heart disease, including scimitar syndrome and atrial septal defect at the age of 50 years. Surgical repair, involving scimitar vein implantation in the left atrium using a pericardial patch, was performed. Despite surgical correction, dyspnea persisted, and hemoptysis developed. A diagnostic workup revealed a critical stenosis of the re-inserted vein. This was successfully treated by percutaneous intervention with stent implantation. The patient has remained asymptomatic since the procedure. Scimitar syndrome can be first diagnosed in adulthood, and clinical manifestations can vary. Diagnostic workup necessitates a CT angiogram, magnetic resonance scan, and catheterization in selected cases. Stenoses of re-implanted pulmonary veins (PVs) can develop years after surgical correction, and hemoptysis may serve as a warning symptom prompting further PV imaging. Percutaneous vascular intervention using a stent is warranted in symptomatic cases and can lead to long-term success.

## Introduction

Partial anomalous pulmonary venous return is a congenital disorder that results in a left-to-right shunt. Scimitar syndrome (SS) represents a variant of partial pulmonary venous return, in which venous blood from the right lung is drained into the inferior vena cava (IVC). Typically manifested in childhood, diagnosis at an adult age is rare. Diagnosis and treatment may be challenging. Universal management strategies have not gained unanimous acceptance. Individual and multidisciplinary team-based decisions are often necessary. Surgical correction is often needed. However, in some cases, surgery can be complicated by symptomatic pulmonary vein (PV) stenosis [1]. In this paper, we present a case of a patient with SS diagnosed at an adult age. This patient underwent surgery that was complicated by symptomatic PV stenosis, necessitating intervention.

## Case presentation

The female patient was born in 1954. As a child, she experienced repeated syncope which was attributed to mitral valve prolapse. At age 47, she first reported undue dyspnea on moderate exertion. Transthoracic echocardiography a year later revealed signs of pulmonary hypertension (PH), along with right ventricular and atrial dilatation. Subsequent transesophageal echocardiography identified an atrial septal defect (ASD).

Consideration for device closure of the ASD led to hospitalization after two years. The patient reported mild physical activity limitation. On auscultation, a low-intensity systolic murmur in the precordium and a split and loud second heart sound over the pulmonic area were noted. Echocardiography indicated physiologic findings on the left heart, mild right ventricular outflow tract dilatation (33 mm), paradoxical septal movement, moderate tricuspid regurgitation, increased peak systolic pulmonary flow velocity (1.5 m/s), and a 10 mm ostium secundum ASD. Catheterization confirmed ASD with a significant left-to-right shunt based on oxygen saturations (Qp:Qs ratio 2:1); measurement of pulmonary artery pressures was not performed. Pulmonary angiography revealed the right upper PV draining into the right atrium.

Subsequently, an attempt was made for surgical closure of the ASD in cardiopulmonary bypass at 28 ºC. It was only during the surgery that the presence of a solitary right PV draining into the IVC was discovered. This single right PV was re-implanted into the left atrium and the ASD was closed, both procedures using a glutaraldehyde-preserved autologous pericardium patch. The patient recovered well and was discharged home on her 20th day postoperatively. However, after the surgery, she remained in functional class II, limited by dyspnea on moderate exercise.

Five years post-surgery, an isolated episode of mild hemoptysis occurred, initially with no further diagnosis. Recurrent hemoptysis in 2014 and 2015 prompted hospitalization. Bronchoscopy revealed no apparent bleeding source but hyperemia in the right-sided bronchial tree regions was noted. Echocardiography showed no significant pathology or PH.

**Figure 1:**
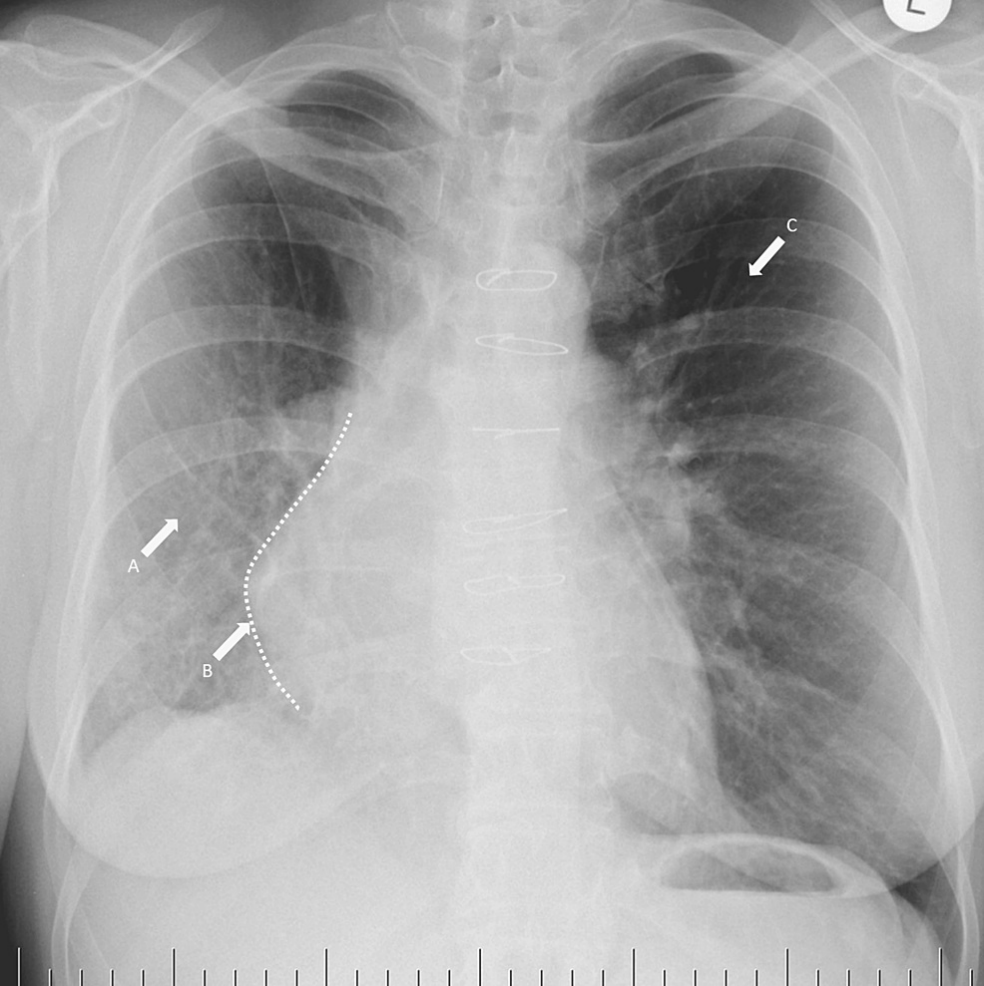
Chest radiograph. Decreased transparency of (A) the right lower lobe, (B) ipsilateral mediastinal shift, and (C) relative hyperlucency of the left upper lobe.

The patient was hospitalized at a tertiary center in 2015. At admission, she presented with no severe complaints. Apart from recurrent mild hemoptysis, she suffered from dyspnea at moderate exercise. She had no angina, no palpitations, no syncope, and no lower limb edema. Her medication included etamsylate, pantoprazole, ezetimibe, escitalopram, and codeine. Her blood pressure was 120/75 mmHg, her heart rate was 84 beats per minute (bpm), and her body mass index was 23.9. There was no remarkable objective finding. Basic laboratory tests, including cardiac troponin, red blood count, and renal functions, were normal, with an NT-proBNP value of 147.6 ng/L. Six-minute walking distance was 550 m. Chest X-ray showed opaque right hemithorax with right-sided mediastinal shift and relative hyperlucency of the left upper lobe due to compensatory left lung hyperinflation (Figure 1).

Further assessments included an electrocardiogram revealing sinus rhythm with incomplete right bundle branch block and a heart rate of 71 bpm. Selected echocardiographic parameters are shown in Table 1.

**Table 1:** Selected transthoracic echocardiographic parameters in 2015. TAPSE, tricuspid annular peak systolic excursion

Parameter		Value
Left ventricle	End-diastolic diameter (mm)	47
	Ejection fraction (%)	60
	Interventricular septal thickness (mm)	8
Left atrium	Diameter (mm)	34
	Volume index (mL/m^2^)	33.5
Right ventricle	Diameter: Basal/middle/base-apex (mm)	35/30/65
	TAPSE (mm)	18
	Fractional area change (%)	28
Pulmonary artery	Time to peak velocity (ms)	125
	Peak systolic velocity (cm/s)	65
Tricuspid regurgitation	Grade	Mild
	Peak systolic gradient (mm Hg)	35
	Inferior vena cava diameter (mm)	15
	Right ventricular systolic pressure estimate (mmHg)	40

**Figure 2:**
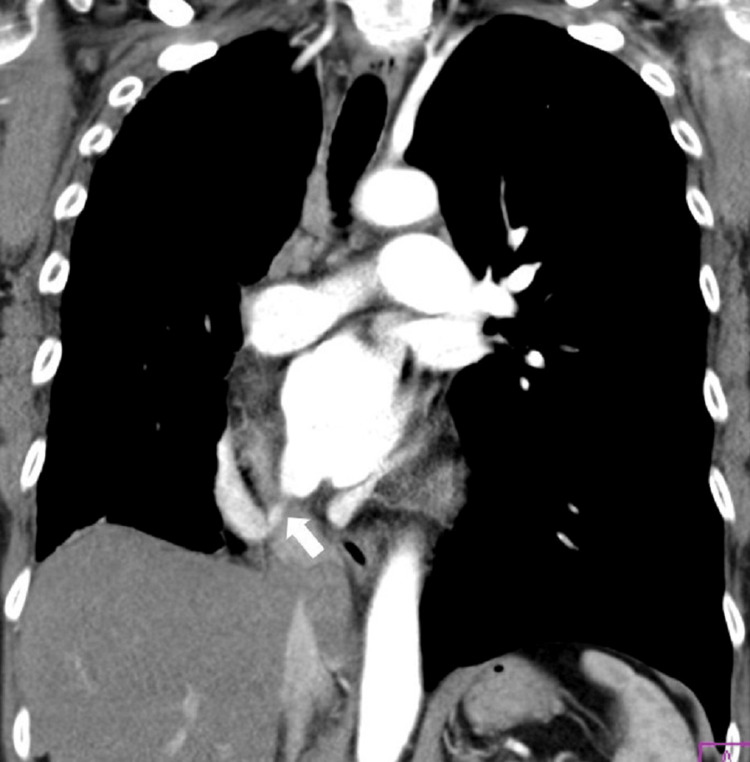
Initial CT image: the re-implanted solitary right pulmonary vein with a critical stenosis at the left atrial confluence (arrow).

No signs indicative of a shunt were observed. CT pulmonary angiography revealed a critical stenosis of the re-implanted single anomalous right-sided PV at the left atrial confluence (Figure 2). Local stasis, hyperemia, and relative hypoplasia of the right lung were present. Left-sided venous drainage was physiological. Pulmonary trunk diameter was 31 mm, right PA measured 23 mm, and left PA measured 25 mm. No signs of chronic pulmonary thromboembolism were identified. Dextroposition of the heart was evident. No residual ASD was present. Additionally, tight concentric stenosis of the IVC was detected just before its right atrial ostium.

Recurrent hemoptysis was attributed to the severe stenosis of the re-implanted single right PV. The heart team decided to pursue a percutaneous intervention using a stent, even in the presence of the pericardial patch in the inter-atrial septum. No specific medical treatment was administered.

**Figure 3:**
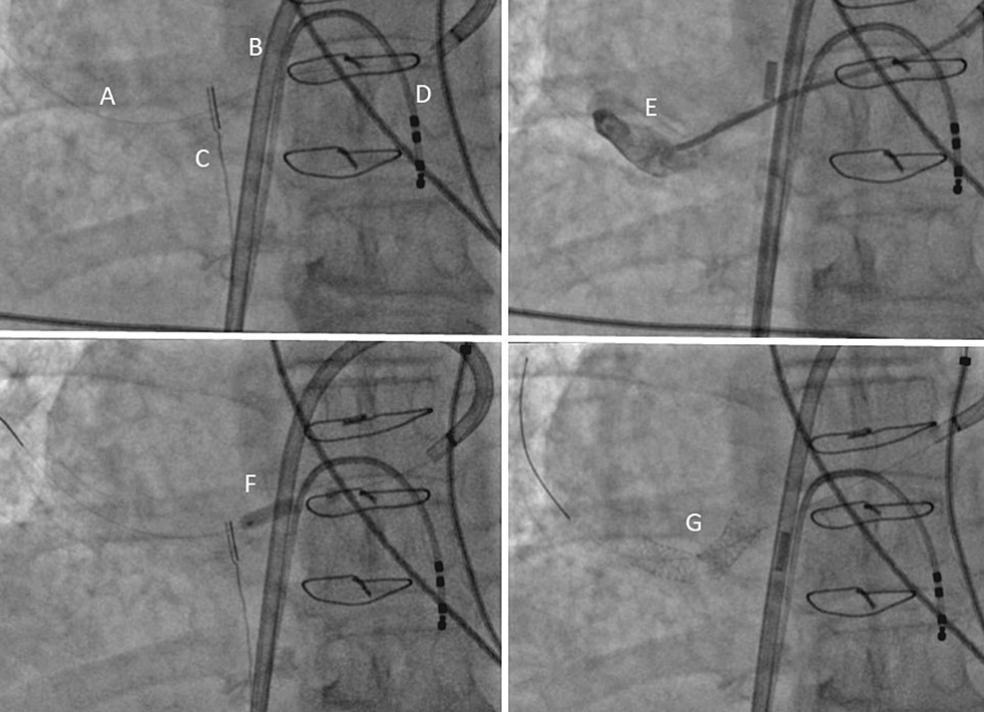
Percutaneous intervention using two stents. (A) Guiding wire in the solitary right pulmonary vein (RPV); (B) steerable sheath trans-septally in the left atrium; (C) intracardiac echocardiography catheter; (D) diagnostic electrophysiology catheter in the right ventricle; (E) angiography of the RPV; (F) inflated balloon in the RPV; (G) two stents in the RPV.

In December 2015, an intracardiac echocardiography-guided trans-septal puncture and subsequent cannulation of the stenotic anastomosis of the common right PV draining into the left atrium were performed. Venous access was established via the right femoral vein, and an 11F sheath was placed for a 10F AcuNav ICE catheter (Biosense Webster, Irvine, CA) and an 8.5 F Agilis NxT steerable introducer (St. Jude Medical, St. Paul, MN). Following trans-septal puncture and venography of the stenotic right PV, pre-dilatation to 4-5 mm was performed, and two stents were implanted yielding a favorable result (Figure 3).

**Figure 4:**
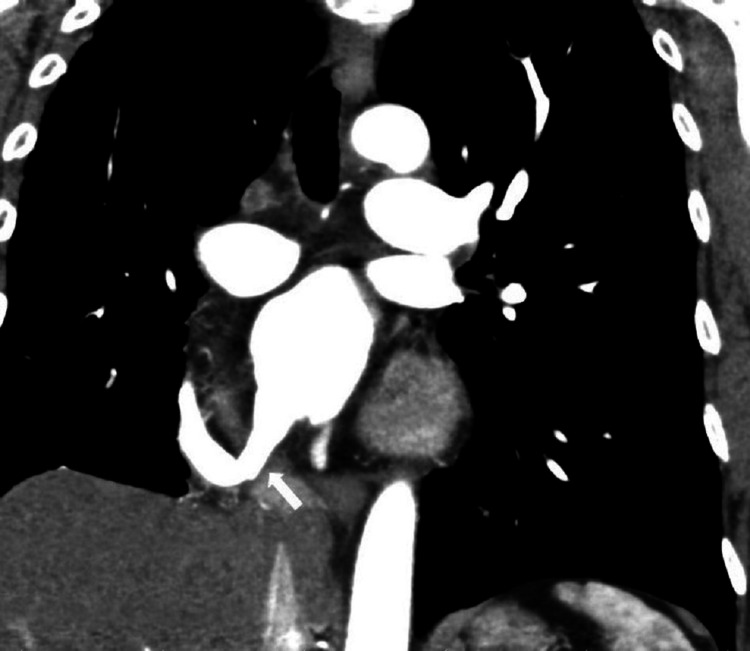
Post-intervention CT image. Solitary right pulmonary vein after dilatation (arrow).

The procedure posed challenges due to severe vascular angulation, but ultimately, two stents were deployed successfully, with a positive angiographic outcome and no peri-procedural complications. Subsequent follow-up CT of the heart and pulmonary venous bed demonstrated the positive effects of the intervention (Figure 4).

Congestive changes in the right lungs diminished, and the patient was discharged home. Dual anti-aggregation treatment was initiated with clopidogrel for three months, along with permanent aspirin use.

**Figure 5:**
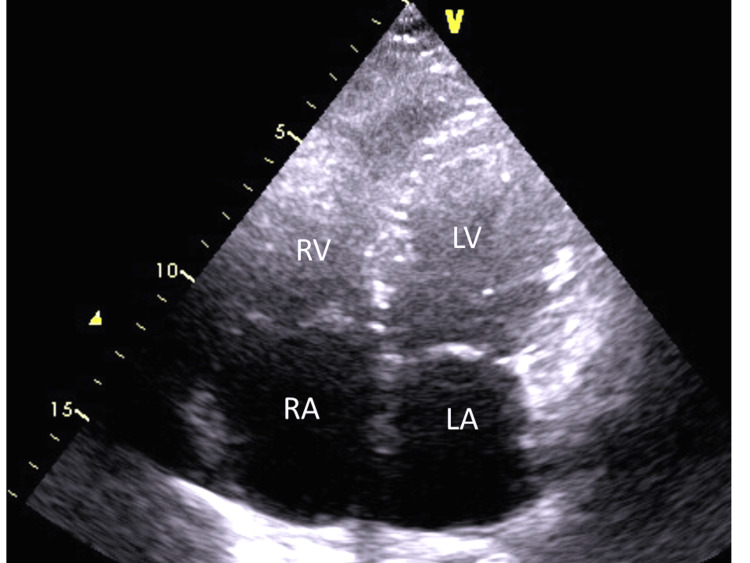
Echocardiogram eight years after the intervention. Apical four-chamber view. RV, right ventricle; RA, right atrium; LV, left ventricle; LA, left atrium

Nine years after the vascular intervention, the patient is classified in functional class I with no other complaints. She is free from hemoptysis, and there are no signs of systemic or pulmonary congestion. Echocardiography reveals a normal-sized right ventricle with good systolic function, mild right atrial enlargement (right atrial area 21.5 cm^2^), and moderate tricuspid regurgitation with a systolic pulmonary pressure estimate of 35 mmHg. Left ventricular morphology and function are normal (Figure 5). Brief timeline of clinical presentation and procedures is summarized in Table 2.

**Table 2:** Case summary. ASD, atrial septal defect; CT, computed tomography; PV, pulmonary vein

Year	Presentation and management
2001-2002	Undue dyspnea: ASD revealed by echocardiography
2004	Surgical ASD closure and single right PV re-implantation into the left atrium
2014-2015	Hemoptysis: significant stenosis of the re-implanted PV revealed by CT pulmonary angiography
2015	Percutaneous vascular intervention on the re-implanted PV
2024	Patient remains asymptomatic

## Discussion

The patient was diagnosed in adulthood, highlighting the atypical presentation of SS. In a retrospective multi-center European study, 19% out of 485 patients were diagnosed at the age of 10 years or older. However, the median age and proportion of adult patients were not specified [1]. According to another recent retrospective single-center study, including patients newly diagnosed with SS in 21 years, 16 out of 47 patients diagnosed in adulthood, with a median age in the adult subgroup of 21.6 years. In the majority of patients, SS was isolated. ASD was present in 3 of the 16 patients diagnosed in adulthood, and 21% of these patients showed concurrent right PA hypoplasia [2]. SS can even be present in the seventh or eighth decade of life with symptoms of heart failure [3,4]. In our case, it is, however, possible that syncopes in childhood were manifestations of SS that were overlooked. Symptoms compatible with heart failure, PH, or shunt (dyspnea on exertion) in our patient first developed at more than 45 years. Also, in the aforementioned cohort of Wang et al., 53.8% of adult patients with isolated SS experienced heart failure and the median age at heart failure onset was 37.7 years. The rest of the adult patients were asymptomatic at diagnosis or had other symptoms [2]. The manifestation of SS in adults can also include some other forms, such as chest pain, palpitations, or hepatomegaly [5,6]. Patients can also be first diagnosed by PH resistant to medical treatment [7]. In asymptomatic patients, the diagnosis is prompted by other findings and associated conditions, such as dextrocardia [8].

The management of this patient involved multiple steps. No CT pulmonary angiography was performed, and the initial diagnosis of anomalous pulmonary venous drainage to the right atrium was concluded after contrast imaging of the right pulmonary artery. It was only during the surgery for associated ASD that the true nature of SS was discovered. Subsequently, anastomosis of the scimitar vein and left atrium and ASD closure, both using pericardial patches, were performed after per-operative consensus of children and adult cardiac surgeons. This mode of repair was prompted by a relatively remote location of the scimitar vein confluence into the IVC.

Currently, the indications of surgery for isolated SS patients manifesting in late adulthood remain less clear with relatively favorable outcomes irrespective of surgical versus medical management [2]. Furthermore, the outcomes for surgical versus nonsurgical management are difficult to compare due to a limited number of reported cases and additional factors [9]. Surgical intervention in adults is recommended for symptomatic patients with pulmonary over-circulation or when the pulmonary-to-systemic flow ratio is greater than 1.5 in asymptomatic patients [9,1]. In our case, the patient was symptomatic, and the Qp:QS ratio was 2:1. However, the assessment of pulmonary versus systemic flow was probably influenced by the presence of ASD. There were no major complications during surgery or the early postoperative period. At the time of surgery, there was no scimitar vein stenosis or obstruction confirmed by periprocedural echocardiography. The overall mortality risk of SS surgical repair is between 4.8% and 5.9%, with patients undergoing correction being at considerably lower risk in comparison with those requiring resective procedures [9]. In a retrospective European study, including children and adult patients, risk factors for hospital and follow-up mortality included lower age, presence of associated congenital heart disease, PH, cardiac symptoms, and necessity for resective procedure as opposed to corrective surgery [1]. In the study by Wang et al., no deaths occurred in the 6 isolated adult patients who underwent surgery during a median follow-up period of 3.9 years [2]. In general, the results of surgery are good and the majority of surviving patients are asymptomatic after surgery [1].

Despite successful surgery, our patient remained in functional class II, and her previous complaints persisted. The time of PV stenosis development is debatable. Hemoptysis as a probably more specific sign of PV stenosis first occurred in 2009. The recurrence of hemoptysis prompted the further diagnostic process as late as 2014. Only after a negative bronchoscopy, further studies including cardiology and imaging were performed and they revealed a critical re-implanted scimitar vein stenosis. Hemoptysis may be common in patients with pulmonary venous stenosis and may be deleterious [10]. According to a meta-analysis, including patients with PV stenoses after PV isolation due to atrial fibrillation, hemoptysis was present in 8% to 63% of patients [11]. Other pulmonary venous stenosis symptoms may include tussive irritation, exertional dyspnea, and recurrent pulmonary infections [10]. Given the history of the patient, hemoptysis should probably have prompted focused imaging in this patient, leading to an earlier diagnosis.

Stenosis of the SS drainage is a recognized long-term complication after correction, posing a challenge after surgery [9]. In a multi-center European study, stenosis/occlusion of the scimitar drainage after corrective procedures in a mixed population of children and adults was present in 25% of patients and was unrelated to the type of corrective technique used [1]. PV stenoses were less frequent in adolescents or adults (14%) than in neonates/infants and children (33% and 20%, respectively). Forty-two patients of all ages with PV stenoses (67%) underwent re-operation or hemodynamic intervention at a median of 0.8 years after repair. Twelve percent of these patients died during the later course. In 60% of surviving patients, the stenosis was asymptomatic and 29% of patients had symptoms [1]. Existing obstruction and distantly located scimitar veins are predisposing factors for PV obstruction development [9]. In children, the sutureless repair is associated with less restenosis and less reintervention [12]. The situation can be different in a pure adult cohort; as in the adult subgroup in a single-center study, the post-repair scimitar stenosis developed in four (67%) of six patients and the median time to post-repair stenosis time was 3.35 years [2]. Symptoms, including persistent dyspnea and recurrent respiratory infections, can be documented in 15% of surviving patients 4.5 years after surgery [9]. Robust data from the adult population with PV stenoses after SS repair are lacking. Some evidence can be derived from a larger number of patients with PV stenoses after PV isolation due to atrial fibrillation. A recent meta-analysis compared balloon angioplasty with stent implantation and included 188 such patients with 315 PV stenoses. After a median follow-up of 32 months, the overall incidence of restenosis was 46%. Compared to stent implantation, percutaneous therapy with balloon angioplasty was associated with a higher risk for restenosis. Procedure-related complications were comparable between balloon angioplasty and stenting [11].

In the presented case, it was decided by the heart team to indicate a percutaneous intervention on the stenotic re-implanted scimitar vein, even in the presence of a pericardial patch in the inter-atrial septum.

The CT-documented IVS stenosis likely developed due to IVS injury at the site of the surgical excision of the scimitar vein. As the patient did not exhibit any symptoms and there were no physical or echocardiographic signs of IVC stenosis or impaired right ventricular filling, the IVC stenosis remained untreated. The patient has not developed signs of IVC stenosis nine years after the vascular intervention.

## Conclusions

We present a case of a patient with complex congenital heart disease, including partial anomalous PV drainage, ASD, and dextroposition of the heart, diagnosed in adulthood. Identification of the true nature of PV drainage to the IVC diagnosis was delayed. Symptoms persisted after the surgical correction. Diagnostic workup due to nonspecific symptoms and hemoptysis revealed a critical stenosis of the re-inserted vein. This was treated by a successful percutaneous intervention. The patient has been asymptomatic since the procedure.

In conclusion, our case highlights the challenges in diagnosing and managing SS, especially when presenting in adulthood. Diagnostic workup necessitates a CT angiogram, magnetic resonance scan, and catheterization in selected cases. The choice between surgical and percutaneous interventions should be individualized, considering the patient's symptoms and anatomical factors. Surgery is warranted for symptomatic patients and those with pulmonary over-circulation or those with a high pulmonary-to-systemic flow. Timely and thorough diagnostic follow-up is crucial for identifying and addressing complications such as PV stenosis. Stenoses of re-implanted scimitar PV can develop years after surgical correction, and hemoptysis can be a warning symptom. Percutaneous vascular intervention with stent implantation can be considered in symptomatic cases and can lead to long-term therapeutic success.
